# Preoperative Super-Selective Embolization versus “On-Clamp” Laparoscopic Partial Nephrectomy for T1 Renal Tumors— A Prospective Randomized Study

**DOI:** 10.15586/jkcvhl.v11i2.328

**Published:** 2024-05-21

**Authors:** Vivek Kumar Singh, Debanga Sarma, Sushant Agarwal, Puskal Kumar Bagchi, Mandeep Phukan, Nabajeet Das, Sasanka Kumar Barua

**Affiliations:** 1 Department of Urology and Renal Transplantation, Gauhati Medical College, Guwahati, Assam, India;; 2 Department of Radiodiagnosis, Gauhati Medical College, Guwahati, Assam, India

**Keywords:** laparoscopic partial nephrectomy, preoperative embolization, radical nephrectomy, renal cell carcinoma, warm ischemia time

## Abstract

To analyze and compare the intraoperative and post-operative outcomes of “on-clamp” laparoscopic partial nephrectomy (LPN) with “preoperative super-selective angioembolization” before LPN. This randomized clinical study was conducted at Gauhati Medical College Hospital, Guwahati, India, between November 2021 and November 2023. Adult patients of either gender diagnosed with T1 renal tumors were included in the study. All patients underwent diethylenetriamine pentaacetate scan preoperatively and at 1-month follow-up. The patients were randomized using a parallel group design with an allocation ratio of 1:1 to receive either preoperative angioembolization followed by LPN or conventional “on-clamp” LPN. Demographic and baseline parameters were recorded along with pre- and post-operative data. There was no significant difference between the two groups in terms of age (P = 0.11), gender distribution (P = 0.32), body mass index (P = 0.43), preoperative hemoglobin (P = 0.34), and preoperative estimated glomerular filtration rate (eGFR; P = 0.64). One patient in the embolization group required radical nephrectomy because of accidental backflow of glue into the renal artery during embolization whereas four patients required clamping due to inadequate embolization. Preoperative super-selective embolization yielded significantly less blood loss, compared to “on-clamp” LPN (145 [50.76 mL] vs. 261 [66.12 mL], P < 0.01). There was no significant difference between post-operative eGFR (at 1 month) between the two groups (P = 0.71). Preoperative embolization offers improved outcomes in the dissection plane, total operative time, and blood loss, compared to conventional “on-clamp” LPN but has no significant effect on change in eGFR.

## Introduction

Renal cell carcinoma (RCC) has been historically treated with radical nephrectomy, while tumors in a single kidney or carcinoma associated with chronic renal insufficiency was treated with nephron-sparing surgery. Numerous studies conducted over the past two decades demonstrated that cure proportions of smaller lesions treated with nephron-sparing surgery were similar to those treated with radical surgery (1, 2). Based on these findings, the latest recommendations also expanded to partial nephrectomy for unilateral small lesions in patients with healthy contralateral kidneys. Laparoscopic renal surgery evolved after Clayman et al. performed the first laparoscopic nephrectomy in 1991 (3). It now includes partial nephrectomy, which has the triple advantage of being minimally invasive while yielding comparable oncological outcomes with acceptable morbidity, and preserving maximal residual kidney function (4, 5). However, laparoscopic partial nephrectomy (LPN) is still a technically challenging procedure, and lack of a perfect method for achieving hemostasis remains a major problem.

Because hypothermia is difficult to achieve laparoscopically, traditional LPN has relied on clamping of renal vessels intraoperatively to aid dissection. Clamping of renal vessels is fraught with its own problems, including ischemia-induced renal parenchymal injury (6).

Transarterial embolization (TAE) of renal tumors was first described in 1973 as a preoperative aid to the resection of localized renal tumors and a means to palliate the clinical manifestations of a metastatic disease (7). Kalman and Varenhorst reviewed published series and demonstrated that preoperative embolization could be used to reduce the size and vascularity of renal tumors, thus providing a mechanical advantage for any subsequent nephrectomy (8). However, because of selection bias and a high occurrence of post-embolization syndrome, the results of studies that assessed the effect of renal embolization on perioperative bleeding in radical nephrectomies have so far produced conflicting (9, 10).

This study attempts to analyze and compare the intraoperative and post-operative outcomes of preoperative super-selective angioembolization before LPN.

## Materials and Methods

### Study Design

**Figure 1:**
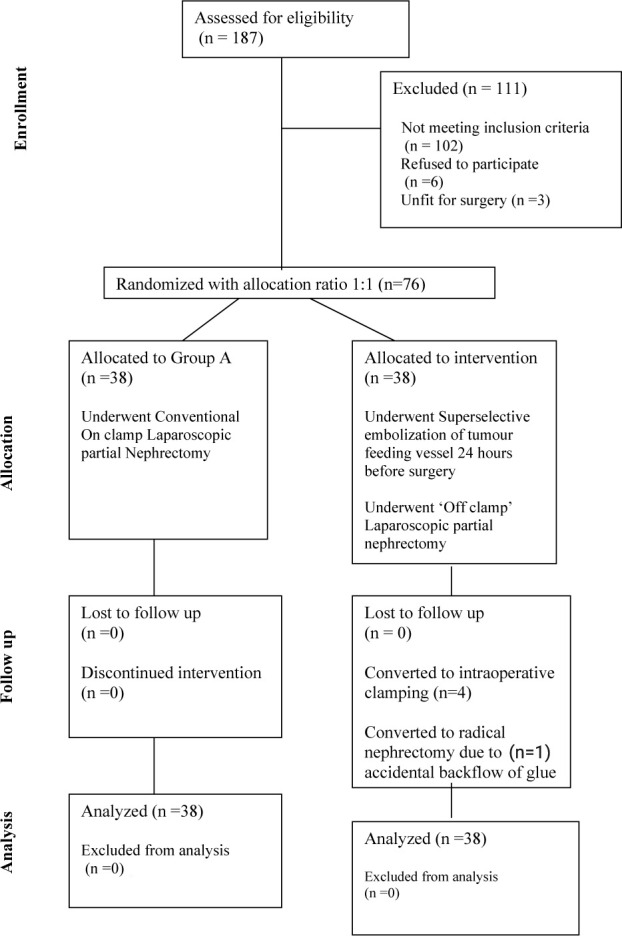
Consort diagram showing patient flow throughout the study.

The study was designed as a single center prospective randomized control trial. Its primary objective was to investigate the effect of preoperative renal mass embolization in terms of total operative time (OT), amount of blood loss, change in estimated glomerular filtration rate (eGFR) after surgery, and complication rate in patients undergoing LPN, and compared it with conventional on-clamp LPN. Our secondary objective was to evaluate the factors affecting post-operative renal function in patients operated with partial nephrectomy. We followed the Consolidated Standards of Reporting Trials (CONSORT) guidelines for reporting the study. The flow of participants through each stage of randomized controlled trial is depicted in Figure 1.

### Ethical Considerations

Institutional ethics committee approval was taken and the trial was registered on clinical trial registry of India (CTRI/2023/09/057607). Written informed consent was obtained from each patient prior to enrollment in the study.

### Inclusion and Exclusion Criteria

All patients presenting at urology outpatient department (OPD) between November 2021 and November 2023 and diagnosed with T1 renal tumor (<7 cm) on imaging investigations (ultrasonography [USG], computed tomography [CT], etc) were included in the study. CT renal angiography was conducted to assess vascular structure of the kidney and identify tumor feeding vessels.

Patients with a tumor size of >7 cm, those who were unfit for surgery, patients with contrast allergy, and those who did not give consent to participate in the study were excluded from the study. All the patients meeting inclusion criteria underwent renal scintigraphy (renal scans: diethylenetriamine pentaacetate [DTPA]/mercaptuacetyltriglycine [MAG3]) to evaluate preoperative eGFR and split renal function; patients also underwent renal scans at 1 month after surgery to look for post-operative eGFR and split renal function.

### Data Collection and Statistics

A fact sheet was prepared containing information about relevant parameters. The content validity was verified by a team of experts from the Department of Urology, Gauhati Medical College Guwahati, India. These patients were randomly allocated to two groups (A and B) using a parallel group design with an allocation ratio of 1:1. An online website-based randomization software was used for the procedure (11). Group A patients were assigned for conventional “on-clamp” LPN. Group B patients underwent preoperative super-selective embolization, followed by LPN.

Based on the study conducted by Benoit et al. (12), and taking mean blood loss in embolization group as 185 mL (SD: 116 mL) and in non-embolization group as 345 mL (SD: 316 mL), sample size was calculated keeping the power as 80%, α at 5%, and allocation ratio as 1. The estimated sample size was 35 patients per group. However, considering the nonresponse and loss of follow up, we considered 38 patients per group as adequate sample size.

The data collected were tabulated and analyzed using STATA version 14. Different statistical aggregates, such as mean (average) values, were used to analyze numerical (scale) variables. Frequency distribution was used in the case of non-numerical (nominal and ordinal) variables. For comparison, Fisher's test and Chi square test were used for categorical data, and Student's *t*-test was used for quantitative variables. Univariate and multivariate logistic regression analysis were used to assess prognostic factors influencing change in eGFR, post-operatively; P < 0.05 was considered statistically significant.

### Embolization Technique

Patients in the angioembolization treatment arm underwent super-selective embolization of renal tumor vessels 24 h prior to surgery at the Division of Interventional Radiology, Department of Radiodiagnosis, Gauhati Medical College, Guwahati, India. Embolization was performed using digital subtraction angiography guidance by an experienced radiologist, and polyvinyl alcohol (PVA) particles (100–300 µm) were used to embolize all vessels supplying the tumor using microcatheter (super-selective embolization). In three cases, acrylic glue was used due to nonavailability of PVA particles. The angiographic endpoints for embolization were complete disappearance of lesion along with the embolization of a rim of healthy parenchyma and complete occlusion of selected feeding vessels.

### Surgical Technique

All surgeries were performed by an experienced urologist with considerable expertise in laparoscopy. LPN was performed using transperitoneal approach, with the patient positioned in the lateral decubitus position. Four trocars were inserted in the case of right renal tumor and three in the case of left renal tumor. After incising the white line of Toldt and reflecting the colon medially, Gerota’s fascia was opened and the kidney was mobilized to identify renal hilum. The renal parenchyma was exposed to isolate the tumor. A 5-mm tumor-free margin outside the boundaries of the tumor was scored, and excision of the tumor was done using scissor. Renorrhaphy was done in two layers using V-Loc (2-0) sutures. Subsequently, hemostasis was secured and specimen removed through one of the ports by extending the incision if necessary and using a specimen-retrieval bag.

### Definitions

The renal score system categorizes renal tumors based on their location relative to polar boundaries. Tumors entirely above or below polar boundaries receive a score of 1. If the tumor crosses the polar line, it gets a score of 2. A score of 3 is assigned if over 50% of the mass crosses the polar line or if the mass is entirely between polar lines. This scoring helps determine tumor complexity: low complexity (score 4–6), moderate complexity (score 7–9), and high complexity (score 10–12) (13).

The classification of recorded complications was made according to the Clavien–Dindo system, which categorizes surgical complications based on their severity (14):

- Grade I: Minor deviations from normal postoperative course.
- Grade II: Complications requiring pharmacological treatment.
- Grade III: Complications necessitating surgical, endoscopic, or radiological intervention without general anesthesia.
- Grade IV: Complications requiring intervention under general anesthesia.
- Grade V: Patient death.

## Results

The study included 76 patients, with 38 in each group randomly assigned to undergo conventional “on-clamp” LPN (group A) and preoperative “super-selective embolization” followed by “off-clamp” LPN (group B). No significant difference was observed between the two groups in terms of age, gender distribution, body mass index (BMI), and preoperative parameters, such hemoglobin, serum creatinine, total eGFR, eGFR of both affected and opposite kidneys, etc (Table 1). Hypertension was the most common comorbidity in both groups (30.5% in group A and 27.7% in group B), while six patients (16.6%) in group A and eight patients (22.2%) in group B were diabetic. One patient in group A had hypothyroidism, hyperparathyroidism with renal calculi, and chronic kidney disease (CKD). In group B as well, one patient had hypothyroidism and CKD.

**Table 1:** Preoperative parameters.

Parameters	Group A (N = 38)	Group B (N = 38)	P value
Age (years)	58.10 (15.03)	54.13 (13.30)	0.11
Gender, n (%)			
Female	22 (57.89)	20 (52.63)	0.32
Male	16 (42.10)	18 (47.36)	
BMI (kg/m^2^)	24.68 (5.15)	25.62 (5.16)	0.43
Surface, n (%)			
Anterior	16 (42.10)	22 (57.89)	0.10
Posterior	16 (42.10)	15 (39.47)	
Both	6 (15.78)	1 (2.63)	
Feeding vessel, n (%)			
Single	21 (55.26)	15 (39.47)	0.25
Multiple	17 (44.73)	23 (60.52)	
Hemoglobin (g/dL)	11.02 (1.39)	10.88 (1.53)	0.34
Creatinine (mg/dL)	0.99 (0.46)	0.94 (0.33)	0.62
Radius (mm)	49.08 (12.79)	49.68 (13.49)	0.42
Diabetes mellitus, n (%)	6 (16.6)	8 (22.2)	0.76
Hypertension, n (%)	11 (30.5)	10 (27.7)	0.99
Side, n (%)			
Left	16 (42.10)	19 (50.00)	0.64
Right	22 (57.89)	19 (50.00)	
Polar location, n (%)			
Upper pole	16 (42.10)	16 (42.10)	0.35
Mid pole	08 (21.05)	05 (13.15)	
Lower pole	14 (36.84)	17 (44.43)	
Preoperative eGFR (mL/min/1.73 m^2^)			
Total	98.35 (14.95)	99.86 (13.46)	0.64
Ipsilateral	45.32 (7.16)	45.34 (7.28)	0.99
Contralateral	52.42 (13.71)	54.52 (12.01)	0.48
RENAL score	6.68 (1.41)	7.18 (1.82)	0.09
RENAL group, n (%)			
Low	19 (50.00)	16 (42.10)	0.13
Moderate	18 (47.36)	16 (42.10)	
High	1 (2.63)	6 (15.78)	

Data are presented as mean (SD) unless otherwise specified.

eGFR: estimated glomerular filtration rate.

### Tumor Characteristics

Mean (SD) tumor size in group A was 49.08 (12.79) mm, compared to 49.68 (13.49) mm in group B. Both groups were comparable in terms of tumor size (P = 0.42). In group A, 42.1% (n = 16) of tumors were faced anteriorly and posteriorly, while 15.8% (n = 6) of tumors reached both anterior and posterior surfaces. While 57.8% (n = 22) of tumors in group B were on anterior surface, 39.4% (n = 15) occupied posterior surface and one tumor reached both surfaces of the kidney.

No significant difference was observed between the two groups on carrying out Fischer’s test (P = 0.10). Mean RENAL nephrometry score (RNS) in both groups was comparable (6.68 in group A vs 7.18 in group B, P = 0.10). In all, 50% (n = 19) patients in group A and 42.1% (n = 16) patients in group B had low complexity tumors (RNS: 4-6), while 47.2% of tumors in group A and 42.1% (n = 16) of patients in group B had moderate complexity tumors (RNS: 6–9).

Regarding severity, 2.6% (n = 1) of tumors in group A and 15.8% (n = 6) of tumors in group B were of complex severity (RNS: 10–12). No statistically significant difference was discovered between the groups on running Chi square test to compare tumor complexity (P = 0.13).

On CT renal angiography, 55.2% (n = 21) of the patients in group A had single feeding vessel supplying renal tumor, while 60.5% (n = 23) of the patients in group B had multiple feeding vessels supplying renal tumor, although this difference was not determined to be statistically significant (P = 0.25). We also observed that tumors that were located centrally had relatively well-defined feeding vessels as compared to peripherally located tumors, which were difficult to embolize due to the presence of en-passage-type feeding vessels.

### Intraoperative Parameters

Preoperatively embolized partial nephrectomy procedures required relatively less time as compared to “on-clamp” LPN (61.47 min in group B vs 79.29 min in group A, P < 0.01). Four patients (10.5%) in group B required intraoperative clamping because of inadequate embolization, while one patient had to undergo radical nephrectomy because of accidental backflow of glue into the main renal artery. Mean warm ischemia time in group A was 24.95 min. Patients in the “embolization” group had significantly less intraoperative blood loss (145 mL), compared to the “on-clamp” nephrectomy group (261 mL; P < 0.01) (Table 2). However, none of the patients in both groups required either intraoperative or post-operative blood transfusion.

**Table 2:** Intraoperative and post-operative parameters.

Parameters	Group A (N = 38)	Group B (N = 38)	P value
Blood loss (mL)	261.05 (66.12)	145.53 (50.76)	<0.01
Operative time (min)	79.29 (18.42)	61.47 (8.12)	<0.01
Conversion, n (%)			
Clamping	NA	4 (10.5)	
Radical nephrectomy	0	1 (2.6)	
WIT (min)	24.95 (4.93)	23.50 (7.78)	
Complications, n (%)			
Clavien–Dindo score 1	4 (10.5)	11 (28.94)	0.04
Clavien–Dindo score 2	7 (18.42)	13 (34.21)	
Clavien–Dindo score 3a	3 (7.89)	2 (5.26)	
Clavien–Dindo score 4	1 (2.6)	0	
Fever	5 (13.15)	11 (28.94)	0.15
Ileus	7 (18.42)	10 (26.31)	0.75
Urine leak	1 (2.6)	0	
Pseudo-aneurysm	1 (2.6)	0	
Surgical site infection	6 (15.78)	8 (21.05)	0.28
Hospital stay (days)	3.1 (1.5)	3.4 (1.7)	0.22
Margin positivity, n (%)	3 (7.89)	1 (2.6)	0.61
Hb (POD 1)	10.45 (1.67)	9.38 (0.97)	
Hb (POD 30)	10.72 (1.68)	9.85 (1.04)	
Hb change (0–30 days)	-0.77 (0.94)	-0.57 (0.70)	0.15
Creatinine change (0–30 days), mean	0.19	0.16	0.83
Post-operative eGFR			
Total	83.44 (16.79)	86.14 (16.70)	0.71
Ipsilateral	27.45 (9.54)	27.63 (10.08)	0.85
Contralateral	55.91 (14.17)	58.59 (12.34)	0.58
Change in eGFR			
Total	14.91 (7.20)	13.72 (7.87)	0.72
Ipsilateral	17.87 (5.52)	17.70 (7.33)	0.58
Contralateral	3.49 (1.99)	4.07 (3.32)	0.11

Data are presented as mean (SD) unless otherwise specified.

WIT: warm ischemia time; Hb: hemoglobin; eGFR: estimated glomerular filtration rate; POD: post-operative day.

### Post-operative Parameters

**Figure 2:**
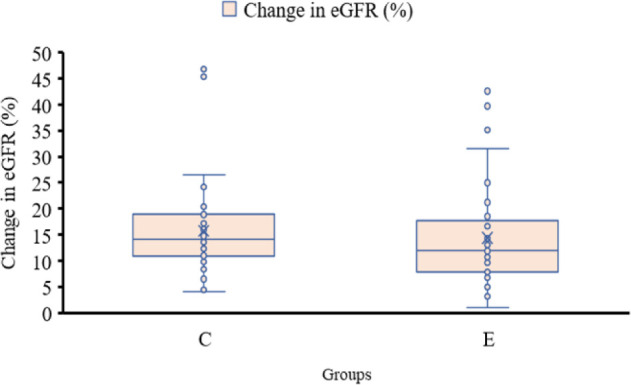
Box and Whisker plot showing percentage change in estimated glomerular filtration rate (eGFR) in both groups.

Mean post-operative eGFR values for groups A and B at 1-month follow-up was 83.44 mL/min and 86.14 mL/min, respectively (P = 0.71) (Figure 2). While mean eGFR (total) dropped by 15.8% (14.91 mL/min) in group A, while in group B, it came down by 14.4% (13.72 mL/min). No statistically significant difference in decline of eGFR (total) was observed between the two groups (P = 0.72). Mean hemoglobin on post-operative day (POD) 1 and POD 30 in group A was 10.45 g/dL and 10.72 g/dL, respectively. In embolization group, mean Hb was 9.38 g/dL and 9.85 g/dL, respectively. Hemoglobin difference in both groups on POD 30 was -0.77 g/dL in group A and -0.57 g/Dl in group B. No clear difference was observed between the two groups on running statistical analysis (P = 0.15).

### Factors Affecting Changes in eGFR (Table 3)

Group A (conventional “on-clamp” LPN): For observations with the actual change in eGFR being <25%, the model correctly predicted all 35 patients, resulting in 100% accuracy.

**Table 3:** Multivariable regression analysis to predict change in eGFR 1 month after LPN.

Conventional “on-clamp” LPN						
	B^*^	Standard error	Wald statistic	Degree of freedom (DoF)	P value	Odds ratio
Age	3.711	286.878	0.000	1	0.990	40.893
Gender	139.340	9734.287	0.000	1	0.989	3.269E+60
BMI	-11.612	945.235	0.000	1	0.990	0.000
RENAL score	20.535	4129.207	0.000	1	0.996	828512187.954
Tumor size	-2.572	220.100	0.000	1	0.991	0.076
Operative time	-2.917	372.808	0.000	1	0.994	0.054
Serum creatinine	-139.044	21661.822	0.000	1	.995	0.000
Preoperative eGFR	-6.762	752.141	0.000	1	0.993	0.001
Constant	848.068	78799.299	0.000	1	0.991	.
Preoperative super-selective embolization followed by LPN						
Age	-0.052	0.107	0.235	1	0.627	0.950
Gender	-0.156	2.777	0.003	1	0.955	0.856
BMI	-0.218	0.254	0.738	1	0.390	0.804
RENAL score	0.729	0.918	0.630	1	0.427	2.072
Tumor size	-0.016	0.100	0.025	1	0.874	0.984
Operative time	-0.018	0.197	0.008	1	0.929	0.983
Serum creatinine	-2.616	3.295	0.630	1	0.427	0.073
Preoperative eGFR	-0.287	0.213	1.811	1	0.178	0.751
Constant	31.612	27.187	1.352	1	0.245	53571434749036.664

* B: coefficient estimates for each predictor variable. These coefficients represent change in the log odds of the outcome for a unit change in predictor variable, holding all other variables constant.

BMI: body mass index; eGFR: estimated glomerular filtration rate.

For observations with the actual change in eGFR being >25%, the model correctly predicted all three patients, also resulting in 100% accuracy. Overall, the model correctly predicted 100% of cases. None of the variables included in the model was a significant predictor for eGFR > 25%.

Group B (preoperative super-selective embolization followed by LPN): For observations with the actual change in eGFR being <25%, the model correctly predicted all 34 cases, resulting in 100% accuracy. For observations with the actual change in eGFR being >25%, the model correctly predicted one out of four cases, resulting in 25% accuracy. Overall, the model correctly predicted 92.1% of cases. None of the variables included in the model was a significant predictor for eGFR > 25%.

### Complications

Higher proportion of complications was observed in the preoperative embolization group, compared to the conventional “on-clamp” LPN group (P = 0.02). However, the majority of post-operative complications in both groups was minor (Clavien–Dindo score 1 and 2) (Table 2). Among major complications (Clavien–Dindo score 3 and 4), one patient required angioembolization for pseudoaneurysm, one required double J (DJ) stenting for urine leak and one required drainage of local wound collection at specimen extraction site in the conventional partial nephrectomy group. Similarly, two patients in the embolization group developed local wound complication requiring secondary suturing under local anesthesia. Most of the surgical site infections (SSI) in both groups occurred at specimen extraction site. One patient in the conventional group required care in intensive care unit (ICU) for septicemia during immediate post-operative period (Clavien–Dindo score 4); however, no mortality was reported in both groups. Mean hospital admission in both groups was the same (group A: 3.1 days, and group B: 3.4 days; P = 0.22).

On histopathological examination, 89.47% of the patients had clear cell carcinoma; two patients had angiomyolipoma, while one patient had papillary carcinoma type 1 and one had Benign renal cyst. Three patients in the conventional group and one in the super-selective group were determined to have unifocal positive surgical margin; however, no recurrence was observed on follow-up in any patient.

## Discussion

Evolvement of renal artery embolization in renal tumors first started in 1969 when Lalli et al. performed transcatheter renal artery embolization in experimental animal models (15). Subsequently various attempts were made and many series were published in the 1970s and 1980s, most notably being the study done by Almgård et al. in 1973, who used autologous muscle to embolize renal artery for preoperative reduction of tumor mass and facilitation of surgery (7, 16). Preoperative embolization is an established treatment in bony metastasis in RCC prior to resection to reduce blood loss (17). Simone et al. were the first to use preoperative super-selective arterial embolization prior to LPN and suggested that it as a feasible alternative to achieve zero ischemia partial nephrectomy (18). In 2009, they published a case series of 110 patients and suggested that this procedure had similar oncologic outcomes as that of open approach with optimal preservation of the renal parenchyma. However, since a healthy rim of normal tissue is also embolized during the procedure, the amount of renal parenchymal loss, compared to conventional partial nephrectomy, has always been a concern. Although there are anecdotal evidences regarding utility of preoperative embolization, its clinical utility is still not widely accepted due to lack of high-powered studies and most of the previous studies were case series or retrospective cohort studies (10). In fact, in a recently published meta-analysis, which screened 13 articles, Shanmugasundaram et al. found only one study that reported comparative outcomes with those who did not undergo preoperative embolization (10). This study conducted by by Benoit et al. utilized hybrid operation theatres and compared LPN with robot-assisted partial nephrectomy (12). While this study was a prospective study, we cannot ignore the fact that use of robotic technology provides improved dexterity and better ease of dissection as compared to conventional LPN and hybrid operation theatres that require considerable financial expenditure.

Previous studies used various methods for estimation of functional outcomes following partial nephrectomy ranging from standard formulas based on serum creatinine values (similar to Modification of Diet in Renal Disease [MDRD]) (19) and CT-based renal volumetry (20) to renal scintigraphy (21). However, use of creatinine-based formulas has their own limitations; for example, MDRD formula is less accurate at eGFR > 60 mL/min (22). Serum creatinine values are also affected by age, gender, muscle mass, and differences in laboratory standards. In fact, about 25% of patients with renal mass having normal serum creatinine and a normal contralateral kidney are found to have CKD (eGFR < 60 mL/min) (23, 24). Use of CT-based renal volumetry probably covers the physiologic effects of renal artery clamping; hence, we used Renal scintigraphy (DTPA scan) to assess both preoperative and post-operative renal functions. Although preoperative embolization leads to absence of vascular clamping and thus the resultant warm ischemia-based renal injury, the risk of embolization of healthy parenchyma leading to significant loss of nephrons was one of the concerns with this procedure. However, we noted that post-operative decline in eGFR was equivalent in both treatment arms (14.91 mL/min in group A and 13.72 mL/min in group B; P = 0.72). It corroborated with the observation made by Benoit et al., who noted that the mean change in eGFR at 1 month was -5.5% in LPN following embolization group versus -8.3% in robot-assisted partial nephrectomy group (P = 0.17) (12).

The present study showed that preoperative super-selective embolization yielded improved operative outcomes as compared to conventional on-clamp partial nephrectomy because of significantly less intraoperative blood loss (145.53 mL vs 261.05 mL), better visualization of resection margin, and comparatively less operative time (61.47 min vs 79.29 min). Results of the present study were similar to the observation made by Shanmugasundaram et al., where preoperative embolization yielded an expected blood loss of 154 mL (n = 222) as compared to 353.4 mL (n = 478) (10). In this meta-analysis, the authors used data from the study conducted by Mir et al., which was a comparative study between radical and partial nephrectomy for T1 tumors; literature search revealed a lack of data showing head-to-head comparison between “on-clamp” LPN and preoperative embolization followed by LPN (25). Total operative time in the study was similar to other studies (20).

Mean warm ischemia time of 24.95 min in the present study was similar to that of other studies published in the literature (26). We also observed that warm ischemia time had no significant effect on change in eGFR postoperatively in multivariate settings (P = 0.14). This finding was consistent with the recent findings of Campbell et al., who observed that warm ischemia during surgery contributed <10% into the post-operative longitudinal atrophy of the kidney (27).

Selective embolization and the resultant thrombosis of feeding vessels also reduce vascular complications following partial nephrectomy, as observed by Shanmugasundaram et al. (10), where none of the patients in embolization arm developed aneurysm or arteriovenous malformation or hemorrhage as compared to 1.7% of patients without embolization. In the present study, one patient in conventional partial nephrectomy developed pseudoaneurysm, while none in embolization arm had this complication. Post-embolization syndrome is commonly characterized by fever, nausea, and pain following embolization. It has been suggested that it can be minimized if surgery is performed within 24 h. However, in this study, 28.9% of the patients in embolization arm developed fever, while two patients complained of access site pain. Both conditions were managed with conservative antipyretic and analgesic medications.

We also observed that tumors, central in location had relatively well-developed feeding vessels whereas tumors located peripherally in the kidney had mostly en passage-type feeding vessels, which were difficult to embolize and resulted in intraoperative loss of plane. In four such patients, we had to use clamp intraoperatively to control bleeding, while one patient in embolization arm had to undergo radical nephrectomy because of accidental backflow of glue into main renal artery.

## Conclusion

The present study demonstrated that the preoperative super-selective embolization of feeding vessels before partial nephrectomy is safe and effective for significantly reducing intraoperative blood loss, with nearly equivalent effect on renal functional outcomes in RCC. However, it takes away the freedom of operating surgeons in deciding the field of resection. It also reduces the risk of major vascular complications, but the procedure may not be successful every time, especially in tumors with en passage-type blood supply.

## Limitations

Limitations of the present study included small follow-up interval of 1 month and small sample size. For conclusive evidence, a large sample size observational study is required to further correlate the relation of tumor vascular pattern with location of tumor.
